# Adoption of Augmented Reality in Educational Programs for Nurses in Intensive Care Units of Tertiary Academic Hospitals: Mixed Methods Study

**DOI:** 10.2196/54188

**Published:** 2024-05-23

**Authors:** Suyoung Yoo, Sejin Heo, Soojin Song, Aeyoung Park, Hyunchung Cho, Yuna Kim, Won Chul Cha, Kyeongsug Kim, Meong Hi Son

**Affiliations:** 1 Department of Digital Health Samsung Advanced Institute for Health Science & Technology Sungkyunkwan University Seoul Republic of Korea; 2 Department of Emergency Medicine Samsung Medical Center Sungkyunkwan University School of Medicine Gangnam-gu, Seoul Republic of Korea; 3 Department of Nursing Education Samsung Medical Center Seoul Republic of Korea; 4 Graduate School of Clinical Nursing Science, Sungkyunkwan University Seoul Republic of Korea

**Keywords:** augmented reality, AR, clinical skills education, nurse education, technology-based education, education, nurse, nursing, allied health, technology-enhanced learning, interview, training, usability, acceptability, educational, teaching, ICU, intensive care unit, self-guided, self-directed, hands-on, adoption, TAM, Technology Acceptance Model, skill, acquisition

## Abstract

**Background:**

In the wake of challenges brought by the COVID-19 pandemic to conventional medical education, the demand for innovative teaching methods has surged. Nurse training, with its focus on hands-on practice and self-directed learning, encountered significant hurdles with conventional approaches. Augmented reality (AR) offers a potential solution to addressing this issue.

**Objective:**

The aim of this study was to develop, introduce, and evaluate an AR-based educational program designed for nurses, focusing on its potential to facilitate hands-on practice and self-directed learning.

**Methods:**

An AR-based educational program for nursing was developed anchored by the Kern six-step framework. First, we identified challenges in conventional teaching methods through interviews and literature reviews. Interviews highlighted the need for hands-on practice and on-site self-directed learning with feedback from a remote site. The training goals of the platform were established by expert trainers and researchers, focusing on the utilization of a ventilator and extracorporeal membrane oxygenation system. Intensive care nurses were enrolled to evaluate AR education. We then assessed usability and acceptability of the AR training using the System Usability Scale and Technology Acceptance Model with intensive care nurses who agreed to test the new platform. Additionally, selected participants provided deeper insights through semistructured interviews.

**Results:**

This study highlights feasibility and key considerations for implementing an AR-based educational program for intensive care unit nurses, focusing on training objectives of the platform. Implemented over 2 months using Microsoft Dynamics 365 Guides and HoloLens 2, 28 participants were trained. Feedback gathered through interviews with the trainers and trainees indicated a positive reception. In particular, the trainees mentioned finding AR particularly useful for hands-on learning, appreciating its realism and the ability for repetitive practice. However, some challenges such as difficulty in adapting to the new technology were expressed. Overall, AR exhibits potential as a supplementary tool in nurse education.

**Conclusions:**

To our knowledge, this is the first study to substitute conventional methods with AR in this specific area of critical care nursing. These results indicate the multiple principal factors to take into consideration when adopting AR education in hospitals. AR is effective in promoting self-directed learning and hands-on practice, with participants displaying active engagement and enhanced skill acquisition.

**Trial Registration:**

ClinicalTrials.gov NCT05629663; https://clinicaltrials.gov/study/NCT05629663.

## Introduction

In recent years, conventional education, especially in the medical field, has been challenged by the introduction of new technologies [1]. The COVID-19 pandemic further highlighted the limitations of conventional teaching methods [2]. Nurse training, with its emphasis on hands-on practice and self-directed learning, was particularly affected by the pandemic, making it evident that conventional training methods could not sustain the demands of the situation [3,4]. Given these constraints, the search for alternative, technology-driven educational methods intensified, aiming to address both physical resource and time challenges without compromising education quality [5,6]. In this context, an immersive learning environment, based on a computer-generated environment enabling real-time user interactions [7], has emerged as a promising solution.

Such an immersive platform merges augmented reality (AR) and virtual reality (VR), offering a dynamic 3D space for learners. This integration not only enhances the learning experience by providing a rich, immersive environment [8,9] but also reshapes the boundaries between reality and the virtual realm, paving the way for innovative learning avenues [10,11]. Exploring AR’s potential reveals that its uses surpass merely aiding in remote education. AR also introduces real-time feedback mechanisms, empowering trainees to obtain immediate insights about their actions and performance through virtual aids [12,13]. This immediacy in feedback is invaluable, as it allows errors to be addressed promptly, fostering continuous improvement in learning [14,15].

In the field of critical care, there has been an exploration of the use of AR and VR in educational applications [16]. VR-enhanced training for tracheostomy care in the intensive care unit (ICU) setting has demonstrated the potential of education in an immersive learning environment [17,18]. Studies have been conducted for training mechanical ventilator settings and central line insertion, showing improvements in self-efficacy, increased familiarity, confidence, and reduced anxiety compared to conventional methods [13,17,19]. However, while the advantages are evident, existing research into AR and VR remains limited. These studies are usually one-time or short-term investigations, mainly focusing on the effectiveness of the immersive learning environment [20]. Moreover, integrating these technologies into a nursing curriculum represents an area yet to be fully explored [21].

In this study, we aimed to derive key considerations for each phase of implementation based on our experience of introducing an AR nursing program within an ICU in a tertiary hospital setting.

## Methods

### Experimental Design

#### Overview

**Figure 1:**
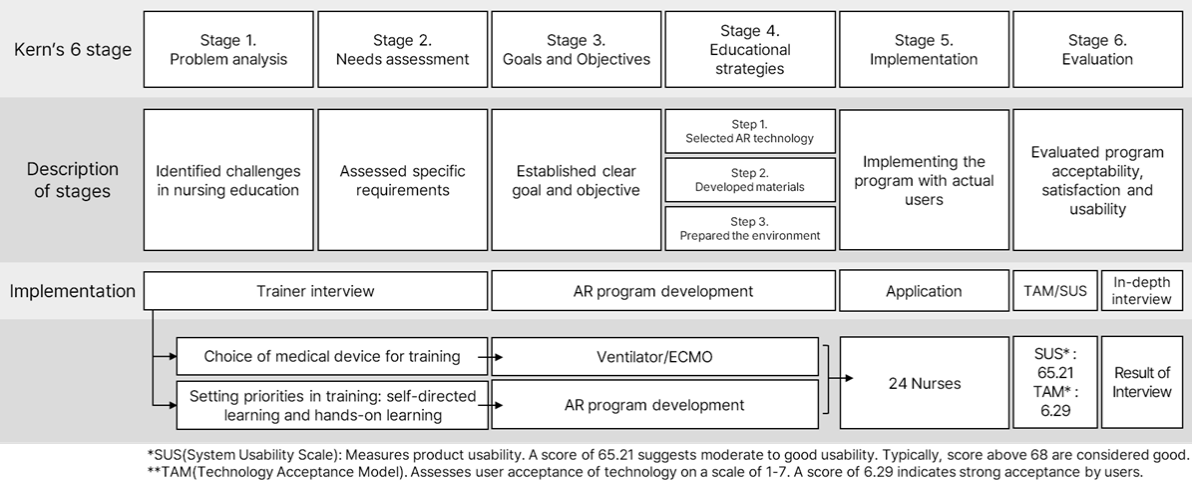
Overall process of the adoption of augmented reality into nursing education based on the Kern 6-step framework [22]. AR: augmented reality; ECMO: extracorporeal mechanical oxygenation.

Our methodology was refined based on the Kern six-step approach to transition nursing education into an immersive AR-based format [22], focusing on the following key stages: (1) problem identification, (2) needs assessment, (3) setting goals and objectives, (4) choosing educational strategies, (5) implementation, and (6) evaluation [23] (Figure 1).

Our initial steps involved conducting interviews with trainer nurses to discern existing issues and identify a procedure amenable to transition into an AR format [24]. We then developed and implemented an AR-based educational program encapsulating two distinct procedures. We surveyed and interviewed trainees, focusing on technology acceptance and usability.

#### Stage 1: Problem Identification

In this foundational stage, we endeavored to identify core problems through a general needs assessment, employing two primary approaches: engaging in interviews with training nurses and reviewing the extant literature [24-27].

The interviews, conducted with nurses from the nursing education department, were based on a semistructured format and were held online or offline depending on participant preference. Voice recordings ensured the precise capture of data shared during these interactions.

In addition to the interviews, we explored previous studies with the aim of harvesting insights and identifying common issues found within the existing research landscape, thereby anchoring our findings in a robust context of existing knowledge [28].

#### Stage 2: Needs Assessment

Upon conducting the interviews in stage 1, we recognized the challenges endemic to conventional educational methods as identified by educators. We investigated possible solutions to these challenges, and found the need for feedback through remote supervision, especially regarding certain devices. We further identified key elements that should be considered in the development of the educational program. This stage also served to validate our problem identification process.

#### Stage 3: Goals And Objectives

We defined our goals through collaboration between trainers and researchers, focusing on improving access and proficiency with complex medical equipment.

#### Stage 4: Educational Strategies

##### Step 1: Selecting Appropriate Technology

With the imperative for hands-on practice in nursing education, AR was chosen to enable nurses to interact with virtual medical devices within a realistic clinical setting [29]. AR’s ability to superimpose digital models onto the physical world allows for a highly interactive and immersive learning experience without the traditional constraints of location, time, or physical resources [30]. This aligns with our goal to empower self-directed learning, permitting nurses to engage in practical education at their own pace and convenience [31].

##### Step 2: Developing Educational Material

Navigating through the lens of self-directed learning and hands-on practice, we considered AR options that could facilitate tangible interaction with 3D objects. The development of materials required a detailed comprehension of the unique needs of nurses and the incorporation of AR content to support self-directed learning.

##### Step 3: Preparation of the Educational Environment

To facilitate a high-fidelity learning experience, our AR-based educational program was set within the hospital’s simulation laboratory. A designated area within the lab was prepared, encompassing a minimum of 3×3 meters to provide trainees with sufficient room to maneuver and interact with the virtual elements without spatial constraints. To ensure uninterrupted delivery of our AR-based educational program, we utilized five HoloLens 2 devices. This approach was adopted to mitigate against battery and overheating issues that could disrupt the learning process. This setup was optimized to allow multiple nurses to receive training at the same time, promoting efficient learning throughout while maintaining an individualized, hands-on experience.

#### Stage 5: Implementation

We recruited the participants through an advertisement posted on the hospital’s internal internet network. Our goal was to enroll a minimum of 10 participants for each session to ensure a dynamic and interactive learning environment while still allowing for personalized instruction. We designed the sessions to accommodate up to five nurses at a time, which was determined as the optimal number for both effective learning and space utilization within our AR setup. This small-group approach not only facilitated focused attention from the instructors but also ensured that each participant could engage deeply with the AR modules.

To maintain a high standard of education and safety, we appointed two experienced supervisors for each training session. These supervisors were selected based on their expertise in intensive care procedures and their familiarity with AR technology. Their role was to provide immediate assistance and feedback, ensuring that any technical issues could be addressed without disrupting the learning process. They were also tasked with observing the sessions to gather informal feedback, contributing to the continuous improvement of the program.

#### Stage 6: Evaluation

This study was conducted at a large academic tertiary hospital in Seoul, Korea, which accommodates more than 3100 nurses and 2000 inpatient beds. The research provided education to intensive care nurses and included a postsession survey and semistructured interview.

### Ethical Considerations

The Institutional Review Board of Samsung Medical Center approved the study design (SMC-2022-08-058 and SMC-2022-08-079), and all trainers and trainees provided written informed consent before participating in the study, ensuring ethical adherence throughout the research. To protect the participants’ privacy and confidentiality, all data collected during this study were anonymized or deidentified. Stringent data protection measures are in place, including the use of secure, encrypted data storage systems accessible only by authorized personnel. These precautions are designed to safeguard sensitive information and maintain the integrity of the data. Participants were compensated for their time and contribution. Each participant received 30,000 KRW (~US $22) upon completion of their involvement in the study. This compensation was intended to acknowledge their valuable time and effort and to offset any inconvenience associated with participation. The compensation structure was clearly communicated to all participants prior to their enrollment and was administered transparently to ensure fairness.

### Outcome Measures of the Survey

Upon completion of the educational program, participants were asked to fill out a questionnaire evaluating their user experience. This evaluation was based on the theories of self-directed learning and hands-on practice, including questions on personal characteristics, job satisfaction, and appropriateness. To evaluate the AR program’s acceptability and usability, we employed the System Usability Scale (SUS) and the Technology Acceptance Model (TAM) [20,32,33].

We chose the SUS for its proven reliability and efficiency across various technologies. The SUS is widely used across various domains, including software, websites, and medical devices, to assess overall user experience. Moreover, it has been validated in hospital environments and shown effectiveness with small sample sizes. The SUS consists of 10 simple questions presented in a 5-point Likert-scale format, assessing both positive and negative aspects of the system, with total scores ranging from 0 to 100 [34,35]. The TAM was selected for its emphasis on understanding user acceptance of information technology [16,36,37]. We adapted the TAM-based survey questions to fit the context of AR nurse education, informed by various relevant studies [24,26,27,38]. In contrast, the SUS was employed in its unmodified form. In addition, we conducted a correlation analysis between the TAM and SUS elements [37,39].

### Outcome Measures of Interviews

Nurses who responded to the questionnaire were selectively screened for their willingness to participate in further interviews. These interviews were semistructured and guided by nursing education theories from previous studies. The format allowed flexibility, permitting up to two additional questions based on the responses of the interviewees.

### Statistical Analysis

The statistical analysis was performed using R software (version 4.3.2). Continuous variables are expressed as either mean (SD) or median (IQR), depending on their distribution, while nominal variables are expressed as counts (n) and percentages (%). We performed a correlation analysis to examine the relationship between the TAM and SUS using survey data.

## Results

### Stage 1: Problem Identification

#### Overview

We obtained interview results from four nurses who are trainers and operators in the nursing education department. The insights garnered from the interviews are summarized below and detailed in Table 1.

**Table 1:** Key considerations in augmented reality (AR) education development as expressed during trainer interviews.

Category	Key details	Core implications
Educational needs and challenges	Training requirements necessary for handling advanced medical equipment; aligning AR educational content to complement the features of specific medical devices; adapting training modules to meet the unique demands precipitated by the COVID-19 pandemic; implementing streamlined training processes for the rapid acclimatization of new nurses; addressing the limitations inherent in conventional training techniques; tackling the deficit of hands-on training equipment in nursing training	Imperative for AR solutions in bridging training disparities and responding to progressive requirements
Program development and strategies	Tailoring educational programs to align with the diverse experience levels of nursing professionals; standardizing the phases of training to ensure uniformity and consistency in educational outcomes; formulating well-structured and strategic plans for nursing training; ensuring efficient and effective distribution and management of training equipment and resources	Criticality of a holistic design and meticulous implementation in AR training for optimal efficacy
Challenges and future concerns	Addressing trainees’ physical challenges, such as the necessity to wear glasses or masks, in the training environment; guaranteeing the safety and appropriateness of both the devices and venues utilized for training; modifying AR training methods to be inclusive and effective for older nursing personnel; integrating strategies within training programs to manage and reduce trainee fatigue effectively	Recognition of and addressing present and prospective hurdles for continuous advancement in AR training

#### Requirement of Education in Difficult-to-Use Devices

Using difficult-to-use medical devices in health care can pose a significant challenge for medical staff due to the increased risk of errors, negatively impacting patient outcomes. Proper education on these devices is essential to ensure that medical staff can use them safely and effectively.

#### Lack of Resources: Space, Instructor, Time, and Cost

The lack of education resources in health care can be a significant challenge for health care organizations and medical staff [15]. Education is essential to ensure that health care professionals possess the necessary knowledge, skills, and abilities to provide safe and effective care to patients. However, the interviewees mentioned that many health care organizations face barriers in providing adequate education resources to their staff, which can negatively impact patient outcomes.

Another challenge is the lack of adequate education time. Health care professionals are often required to work long hours, and finding time to attend education sessions and complete the necessary course work can be difficult. This lack of time can make it difficult for health care organizations to provide education tailored to the specific needs of their staff.

### Stage 2: Needs Assessment

Following the interviews, we identified crucial factors to consider when selecting educational topics. Educators highlighted the significant challenges of limited access to educational devices and instructors. Additionally, they emphasized the necessity for education in technically demanding skills. Trainers expressed a preference for educational topics that required hands-on practice. Their reasoning is grounded in the knowledge that complex devices are frequently used in treating patients with critical illnesses. The competence of nurses in operating these devices directly impacts patient outcomes. The responses from trainers and operators related to needs are summarized in Table 1.

### Stage 3: Goals and Objectives

#### Selected Objectives

Based on the interview results, extracorporeal membrane oxygenation (ECMO) machines and ventilators were selected as the objectives for training owing to their complexity and difficulty of use. Ventilators were selected as important yet challenging devices to master. The complexity of ventilators, compounded by the multitude of lines and connections involved, can pose challenges for nurses with limited experience.

By contrast, an ECMO machine is a high-risk medical device that is essential for patients with COVID-19. When the alarm of an ECMO machine sounds, nurses must promptly find a solution. However, given its rarity, even experienced nurses may not have encountered this situation. Nevertheless, as this could pose a risk to the patient, appropriate education was deemed necessary, and therefore use of the ECMO machine was selected as the problem scenario for this evaluation.

#### Selected Goals

Our aim was to develop a sustainable AR-based educational program that could offer numerous benefits to trainers and trainees. These benefits include enhanced engagement and motivation, interactive and immersive learning experiences, and the facilitation of personalized learning. Key considerations for developing such a sustainable AR-based educational program encompass designing for scalability and accessibility, and incorporating user feedback to enhance the program in its maintenance and operation over time.

### Stage 4: Educational Strategies

#### Step 1: Development of an AR-Based Educational Program

The ECMO machine educational program comprises 45 slides divided into four parts, each detailing the operation of the machine, managing machine disruptions, responding to “low battery” alarms, and addressing the loss of flow signal (“SIG”) alarm. Each part includes approximately 8-10 steps, guiding trainees on how to manage each situation effectively.

The ventilator program is composed of three parts encompassing a total of 46 slides. Each part involves 26 steps related to ventilator settings and preuse inspections, as well as seven steps for application, alarm configuration, and educational content evaluation. The education process involves checking supplies, power sources, wall oxygen and medical gas connections, and exhalation cassette connections; powering on the device; performing preuse checks; connecting test tubes; selecting the target and application method; turning on the humidifier; configuring the mode and parameters; connecting the patient to the system; monitoring after patient application; and setting alarms.

#### Step 2: Adoption of Innovative Technology

The AR-based education was performed with a Maquet Servo-I mechanical ventilator and the RotaFlow II System Permanent Life Support ECMO machine from Getinge. We attempted to incorporate a 3D guide for hands-on practice and used videos to enhance understanding. The AR-based educational program was developed using the Microsoft Dynamics 365 Guides program. The program’s content was delivered to users through a Microsoft HoloLens 2 device.

#### Step 3: Operation Plan

The previously designated simulation laboratory was successfully used during the AR-based educational program. The allocated space for the program proved sufficient, with each trainee having access to the minimum 3×3 meters space as planned [40]. This spatial arrangement allowed for unimpeded movement and interaction with the AR components, which was critical for the immersive learning experience.

In practice, the ventilator and ECMO machine simulations were conducted without any spatial constraints, enabling a total of 28 trainees to complete the training per the session schedules. The effective use of space was evidenced by the trainees’ ability to perform the necessary tasks and their reported comfort level during the training sessions.

### Stage 5: Implementation

The AR-based educational program platform was operational for a period of 2 months, with education sessions scheduled from 9 AM to 5 PM. This schedule allowed nurses to select their preferred date and time within this interval. To facilitate the program’s implementation, we used five HoloLens 2 devices, along with two laptops for supervisor screen connections and two large screens for the research environment. Throughout the research, a total of 22 trainees actively engaged in the education sessions. The trainees’ screens, as viewed through the HoloLens 2 devices, were immediately visible to the trainer, enabling real-time progress monitoring. Additionally, trainees were encouraged to request assistance if they encountered any difficulties during the session.

### Stage 6: Evaluation

#### Participants

Training sessions were conducted by two trainers and two operators for the 28 nurses in the ICU from January 1 to February 3, 2022. Twenty-four nurses participated in the survey, 11 of whom took part in an in-depth interview. They were trained either in ventilator or ECMO machine usage with an even distribution across both groups. The participants’ baseline characteristics are presented in Table 2. The median work experience was 3 (IQR 0-6.25) years with a mean of 3.75 (SD 3.90) years. There was a predominance of female participants (17/24, 71%). All participants belonged to the general nursing field with a slight majority working in the medical ICU compared to the surgical ICU (Table 2).

Additionally, all participants (24/24, 100%) owned smartphones and the majority (23/24, 95.83%) possessed either a tablet PC or laptop. Prior to the instruction, 13 (54%) nurses had previous experience with a head-mounted display.

**Table 2:** Demographic and clinical characteristics of the surveyed nurse trainees (N=24).

Characteristics		Trainees, n (%)
**Method trained on**		
	ECMO^a^	12 (50)
	Ventilator	12 (50)
**Sex**		
	Male	7 (29)
	Female	17 (71)
**Medicine specialty**		
	Internal medicine	15 (63)
	Surgical department	9 (38)
**Experience (years)**		
	<1	5 (25)
	1-2	3 (15)
	3-4	2 (10)
	5-6	2 (10)
	≥7	3 (15)

^a^ECMO: extracorporeal membrane oxygenation.

#### Comparison of SUS and TAM Scores

In the usability test, the items “I think that I would like to use this system frequently” and “I don’t think the system is unnecessarily complex” received the highest rating of 4.38 out of a possible 5, while the lowest-rated item, “I thought there was too much inconsistency in this system,” received an average score of 1.83. The responses concerning technology acceptance were categorized into four areas according to the TAM: perceived usefulness (PU), perceived ease of use (PEU), perceived enjoyment (PE), and intention to use (IU). The survey included 15 questions scored on a 7-point scale. The item with the highest score was “It is fun to use,” scoring 6.71, while the lowest-rated item was “It is easy to use,” scoring 5.17. In further survey results, factors such as age, sex, department of work, and years of work did not impact satisfaction with the education or usability. All response results for the survey are provided in Multimedia Appendix 1.

#### Correlation of Usability and Acceptance

Our correlation analysis revealed varying degrees of association between SUS and TAM factors. For instance, there was a strong correlation between PU and IU and a moderate correlation between PE and PU. However, the correlation between PEU and IU was not significant (Table 3).

**Table 3:** Correlation between the Technology Acceptance Model–based survey items and System Usability Scale (SUS).

Variable			UE^a^ (SUS)		PU^b^		PEU^c^	PE^d^		IU^e^
**UE (SUS)**										
	*r*	1		0.2		0.34		0.15	0.22	
	*P* value	—^f^		.50		.12		.45	.30	
**PU**										
	*r*	0.2		1		0.2		0.69	0.76	
	*P* value	.50		—		.50		<.001	<.001	
**PEU**										
	*r*	0.34		0.20		1			0.31	
	*P* value	.12		.50		—		.34	.33	
**PE**										
	*r*	0.15		0.69		0.32		1	0.72	
	*P* value	.45		<.001		.34		—	<.001	
**IU**										
	*r*	0.22		0.76		0.31		0.72	1	
	*P* value	.30		<.001		0.33		<.001	—	

^a^UE: user experience.

^b^PU: perceived usefulness.

^c^PEU: perceived ease of use.

^d^PE: perceived enjoyment.

^e^IU: intention to use.

^f^Not applicable.

#### Insights From Participant Interviews

Four participants completed interviews related to their experiences with the AR-based educational program. Key insights from these interviews have been collated and are summarized in Table 4. We present a curated selection of interview responses that most effectively capture the key insights. These selections were thoroughly chosen for their relevance and ability to represent the broader findings of the study.

Overall, the evaluation of the AR-based education was positive, with participants indicating that AR could enhance their actual clinical performance. AR technology is particularly well-suited for individuals interested in self-directed or hands-on learning theories. Nurses were found to be open to education using innovative technology. When asked if they needed assistance with the curriculum, no participant responded negatively regarding the content. However, some participants did express a need for help in adapting to new devices and technologies.

**Table 4:** Trainees’ feedback after augmented reality (AR) implementation in the training program.

Category and subcategory			Details
**Motivation**			
	Intrinsic motivation	Interest in the integration of AR into educational settings; desire among learners for practical experience with medical devices; expectations that AR technology will significantly improve learning efficacy	
	Reasons for participation	Influence of colleague recommendations; curiosity about AR teaching methods; specific needs related to own job	
	Existing issues	Noticing varying standards in educational quality; underscoring the need for improvement; necessity to establish standardized training procedures and protocols; need for training programs to be customized to individual learning styles and needs	
**Learning preferences**			
	Preferred learning method	Balancing traditional (54.5%) and self-directed (45.5%) learning approaches; valuing feedback and interaction in traditional learning; preference for flexibility and pace in self-directed learning	
	Face-to-face versus nonface-to-face	Equilibrium between face-to-face (54.5%) and remote learning (45.5%); diverse preferences shaped by feedback, comfort, and flexibility	
**Feedback**			
	Practical use	Majority opinion holding that AR technology is beneficial for skill development; mixed opinions regarding the real-world applicability of AR in professional settings; varied levels of expectation regarding the use of AR devices in educational contexts	
	Training experience	Recognizing the benefits of AR in providing realistic scenarios, allowing for self-directed learning, and enabling repeated practice; challenges include unfamiliarity with AR, focus on operation over content, and limited interaction	
	Content and support	General satisfaction with AR content amid comparisons to traditional methods; requirement for technical support and assistance in AR training	
	Comparative analysis and outlook	AR’s superiority in learning pace, error identification, and training repetition over conventional methods; challenges in mastering AR operation and content depth; mixed perspectives on AR replacing traditional methods (viewed as supplementary); AR’s efficacy in specific scenarios; considered resource-intensive for broad implementation; potential for enhancing self-directed and iterative learning	
**Future considerations**			
	Target demographics for AR training	Target new nurses, individuals lacking device experience, and department transfers; however, limited relevance for experienced nurses	
	Benefits of self-learning with AR	Reduced pressure, time efficiency, review flexibility; utility in learning uncommon scenarios and repeatable sessions	
	Concerns with self-learning	Limitations of AR training in actual clinical settings; lack of communal learning opportunities in AR environments; concerns over system errors and device quantity limitations	

## Discussion

### Principal Results

The principal findings of this study provide valuable insights into the strategic translation of conventional critical nursing education to AR-based education platforms in the use of difficult-to-use medical devices [41]. Through interviews conducted with trainers before program development, the study successfully identified the specific needs and requirements of trainers in critical care nursing education. The study employed AR-based educational technology to enhance self-directed learning and hands-on practice.

In the educational strategies employed, the study leveraged the unique features of AR to facilitate self-directed learning. By offering interactive and self-controlled learning experiences, AR empowered trainees to take ownership of their learning process. The program incorporated instructional materials and modules that allowed learners to explore and acquire knowledge at their own pace, fostering a sense of autonomy and self-guided learning. The use of AR also enabled real-time feedback and assessment, allowing learners to track their progress and identify areas for improvement [29].

Through the overlay of 3D objects and virtual models onto real-world settings, trainees engaged in simulated scenarios closely resembling authentic ICU environments. This hands-on component of the program enabled learners to apply their knowledge and skills in realistic contexts, promoting an understanding of the subject matter and the development of critical thinking and problem-solving abilities.

This study highlights how AR technology significantly contributes to the success of self-directed learning and hands-on practice. The utilization of AR technology facilitates active engagement, learner-centeredness, and skill development, thereby enhancing the overall effectiveness of critical care nursing education. Moreover, we provide useful insights based on the perspectives of trainers and operators of the platform. The inherent nature of education often necessitates a lower number of educators compared to learners. The main strength of our study thus lies in presenting an infrequent perspective of educators, a viewpoint seldom encountered within the large-scale hospital setting.

### Comparison With Prior Work

AR technology has been extensively explored in areas related to nursing education [42] such as surgical simulation [43], anatomy education [20], and patient safety education [44]. However, it is worth noting that this study represents the first investigation into the use of AR for replacing a conventional educational program in the use of difficult-to-use devices such as an ECMO machine and mechanical ventilator specifically within the critical care nursing field. By incorporating AR into these fields, this study pioneers the integration of innovative approaches in nursing education.

### Limitations

This study, being characteristic of a pilot study to identify and apply new educational methods, has the limitation of a restricted number of participants. In further research, a larger sample size could be recruited to identify factors influencing user acceptability and to enhance usability, leveraging insights for more effective implementation.

### Implications

This study highlights the implications of AR in future research and practice. The findings suggest the need for longitudinal studies to assess AR’s long-term impact on clinical performance and patient outcomes, and to explore its scalability and cost-effectiveness compared to traditional training. Practically, the results of our study indicate that institutions adopting AR should invest in technical support and training and consider integrating AR as a supplementary tool in curricula for a blended learning approach.

### Conclusions

This study provides insights on the development, launch, and operation of an AR-based medical educational program. The study suggests that an AR-based educational program can be an alternative to compensate for insufficient resources for conventional critical care nursing education. Further research can be conducted to compare the effectiveness and feasibility of this program with other AR-based educational programs and traditional nursing educational programs.

## Appendix

Multimedia Appendix 1 All response results for the System Usability Scale and survey based on the Technology Acceptance Model.

## Abbreviations

AR: augmented reality
ECMO: extracorporeal membrane oxygenation
ICU: intensive care unit
IU: intention to use
PE: perceived enjoyment
PEU: perceived ease of use
PU: perceived usefulness
SUS: System Usability Scale
TAM: Technology Acceptance Model
VR: virtual reality
