# Supplementary material for: Adoption of Augmented Reality in Educational Programs for Nurses in Intensive Care Units of Tertiary Academic Hospitals: Mixed Methods Study
Source: JMIR Serious Games. 2024 May 23;12:e54188. doi: 10.2196/54188 (PMC11157172; doi:10.2196/54188)
Supplement: Multimedia Appendix 1 [file games_v12i1e54188_app1.docx]

Multimedia Appendix 1. All response results for System Usability Scale and Technology Acceptance Model

| System Usability Scale | | | |
| --- | --- | --- | --- |
| Variable | Mean | Median  (Q1, Q3) | Min, Max |
| Q1 I think that I would like to use this system frequently. | 4.38 | 5 (4, 5) | 3, 5 |
| Q2 I found the system to be easy to use. | 4.38 | 5 (4, 5) | 2, 5 |
| Q3 I think that the various functions in this system are well integrated. | 3.92 | 4 (3, 5) | 1, 5 |
| Q4 I had to learn a lot of things before I could get going with this system. | 3.79 | 4 (3.75, 4.25) | 1, 5 |
| Q5 I found the system unnecessarily complex. | 4.33 | 4 (4, 5) | 3, 5 |
| Q6 I thought the system was easy to learn. | 1.83 | 2 (1, 2) | 1, 5 |
| Q7 I found the system cumbersome to use. | 4.13 | 4 (3.75, 5) | 3, 5 |
| Q8 I felt very confident using the system. | 2.25 | 2 (1, 3) | 1, 5 |
| Q9 I needed to learn a lot of things before I could get going with this system. | 4.08 | 4 (4, 5) | 2, 5 |
| Q10 I would imagine that most people would learn to use this system very quickly. | 2.5 | 2 (2, 3.25) | 1, 5 |

| Technology acceptance model | | | |
| --- | --- | --- | --- |
| Variable | Mean | Median  (Q1, Q3) | Min, Max |
| PU (Perceived Usefulness) | | | |
| Q1: The use of Hololens 2 would enhance learning abilities and proficiency in the respective educational program. | 6.29 | 6.5 (6, 7) | 4, 7 |
| Q2: Using Hololens 2 during education makes it easier to understand important concepts. | 6.08 | 6.5 (6, 7) | 3, 7 |
| Q3: I believe Hololens 2 is useful as an educational tool. | 6.29 | 7 (6, 7) | 3, 7 |
| Q4: Utilizing Hololens 2 would improve the quality of my actual skill performance. | 6.17 | 6.5 (6, 7) | 3, 7 |
| PEU (Perceived ease of Use) | | | |
| Q5: Hololens 2 is easy to use. | 5.17 | 5 (4, 6) | 3, 7 |
| Q6: Learning how to use Hololens 2 was not difficult. | 6.21 | 6 (6, 7) | 3, 7 |
| Q7: Learning how to use Hololens 2 was clear and easy to understand. | 6.38 | 6.5 (6, 7) | 5, 7 |
| PE (Perceived Enjoyment) | | | |
| Q8: Using Hololens 2 is enjoyable. | 6.71 | 7 (7, 7) | 4, 7 |
| Q9: I enjoyed using Hololens 2. | 6.58 | 7 (7, 7) | 3, 7 |
| Q10: I believe I can learn through Hololens 2 in a similar way as playing a game. | 6.54 | 7 (6.75, 7) | 4, 7 |
| IU (Intention to Use) | | | |
| Q11: The use of Hololens 2 makes learning more engaging. | 6.5 | 7 (6, 7) | 3, 7 |
| Q12: I did not find it boring while using Hololens 2. | 6.63 | 7 (6.75, 7) | 5, 7 |
| Q13: I think it would be beneficial to use Hololens 2 in a hospital setting. | 6 | 6.5 (65 7) | 3, 7 |
| Q14: If given the opportunity, I would like to use Hololens 2 in the future. | 6.29 | 7 (6, 7) | 4, 7 |
| Q15: I would like to use Hololens 2 to learn different skills or fields. | 6.54 | 7 (6, 7) | 4, 7 |
